# Survival Characteristics and Transcriptomic Analyses Reveal the Adaptive Response of the Aquatic Pathogen Non-O1/O139 Vibrio cholerae to Starvation Stress

**DOI:** 10.1128/spectrum.01939-21

**Published:** 2022-05-09

**Authors:** Xiaojian Gao, Zirui Zhang, Qieqi Qian, Qiyun Chen, Shuwen Gu, Jie Li, Yingjie Zhang, Congcong Wu, Qun Jiang, Xiaojun Zhang

**Affiliations:** a College of Animal Science and Technology, Yangzhou University, Yangzhou, China; State Key Laboratory of Microbial Resources, Institute of Microbiology, Chinese Academy of Sciences

**Keywords:** non-O1/O139 *V. cholerae*, starvation, survival, transcriptome, stress responses

## Abstract

Non-O1/O139 Vibrio cholerae is a pathogen of various aquatic organisms but requires major self-regulation to overcome environmental stress in the aquatic environment. However, its survival strategies under environmental stress are not well understood. The objective of this study was to describe the survival characteristics and changes in expression of stress resistance-related genes of non-O1/O139 V. cholerae after 6 months of starvation at room temperature. The results demonstrated that starved cells were still viable, exhibited shortened rods and shrinking surface, and maintained virulence to Macrobrachium rosenbergii. To investigate the changes in gene expression in non-O1/O139 V. cholerae under starvation stress, especially those involved in stress resistance, transcriptome profiles of starved and wild-type cells were determined. The differentially expressed genes (DEGs) in starved cells were identified, including 191 upregulated genes and 180 downregulated genes. Among these DEGs, the well-known stress resistance-related genes were upregulated significantly, including *rpoS*, *rpoD*, *rpoN*, *rpoE*, *uspA*, *uspC*, *cspD*, *hslJ*, etc. Gene Ontology (GO) analysis of the DEGs demonstrated that environmental adaptation-related categories, such as response to stimulus and signal transduction, were upregulated significantly in the starved cells, while cell motility was downregulated significantly. These DEGs were also enriched into 54 KEGG (Kyoto Encyclopedia of Genes and Genomes) pathways, including biofilm formation, two-component system, quorum sensing, flagellar assembly, bacterial chemotaxis stress resistance-related pathways, etc. The potential existence of long-starved non-O1/O139 V. cholerae bacteria in the aquatic environment may raise new concerns about this devastating pathogen in aquaculture.

**IMPORTANCE** Non-O1/O139 V. cholerae is a causal agent of vibriosis that can be subject to nutrient insufficiency and cause high rates of mortality in aquatic animals. However, its molecular mechanisms of survival in response to starvation stress have been investigated only partially. Here, we demonstrate that under starvation stress, non-O1/O139 V. cholerae can survive over the long term and cause disease by dwarfing of the cell structure, upregulation of a series of stress resistance-related genes, and downregulation of flagellum assembly-related genes. This knowledge can help the development of intervention strategies to control non-O1/O139 V. cholerae infection in aquaculture.

## INTRODUCTION

Vibrio cholerae is a Gram-negative, motile, rod-shaped bacterium that inhabits various aquatic environments, including marine and freshwater environments (1). More than 200 serogroups of V. cholerae have been identified, and only serogroups O1 and O139 have been well documented to be involved in the outbreak of epidemic cholera (2). In recent years, non-O1/O139 V. cholerae have been identified as pathogens of many aquatic animals, such as Macrobrachium rosenbergii (3), Macrobrachium nipponense (4), Penaeus monodon (5), and Cyprinus carpio (6). In response to the significant losses caused by non-O1/O139 V. cholerae to the aquaculture industry, most studies on V. cholerae have focused on the pathogenesis of this bacterium, as well as detection and prevention strategies. However, the survival strategies of non-O1/O139 V. cholerae under environmental stress are not well understood.

Bacteria in natural environments are usually challenged by various stressful conditions, including starvation, temperature, pH, oxygen, and osmolality pressure (7). Usually, lack of nutrients is the most common environmental stress that microorganisms routinely encounter in natural ecosystems (8). However, it was found that Vibrio spp. can survive for a long time during starvation by sequential changes in cell physiology and morphology (8, 9). The first noticeable change of these Vibrio spp. is dwarfing of the cell structure under starvation stress, which is beneficial by minimizing the requirements for cell maintenance and adaptation to environmental stresses. Moreover, it was reported that some species would enter a viable-but-nonculturable (VBNC) state in response to starvation stress (10). In addition, Vibrio spp. will adjust their gene expression (e.g., sigma factors, virulence-related genes, and metabolism-related genes) to adapt to starvation stress (11). Although some environmental factors affecting V. cholerae physiology have been reviewed (12), molecular-level responses of non-O1/O139 V. cholerae from diseased aquatic animals under prevailing starvation conditions are still scarce.

In this study, we focused on the changes in the morphology, survival, virulence characteristics, and expression of stress resistance-related genes of non-O1/O139 V. cholerae strain GXFL1-4 cells under starvation stress. This study will contribute to understanding the regulatory mechanisms of non-O1/O139 V. cholerae cells in favoring survival under starvation stress.

## RESULTS

### Survival of non-O1/O139 V. cholerae under starvation stress.

**FIG 1:**
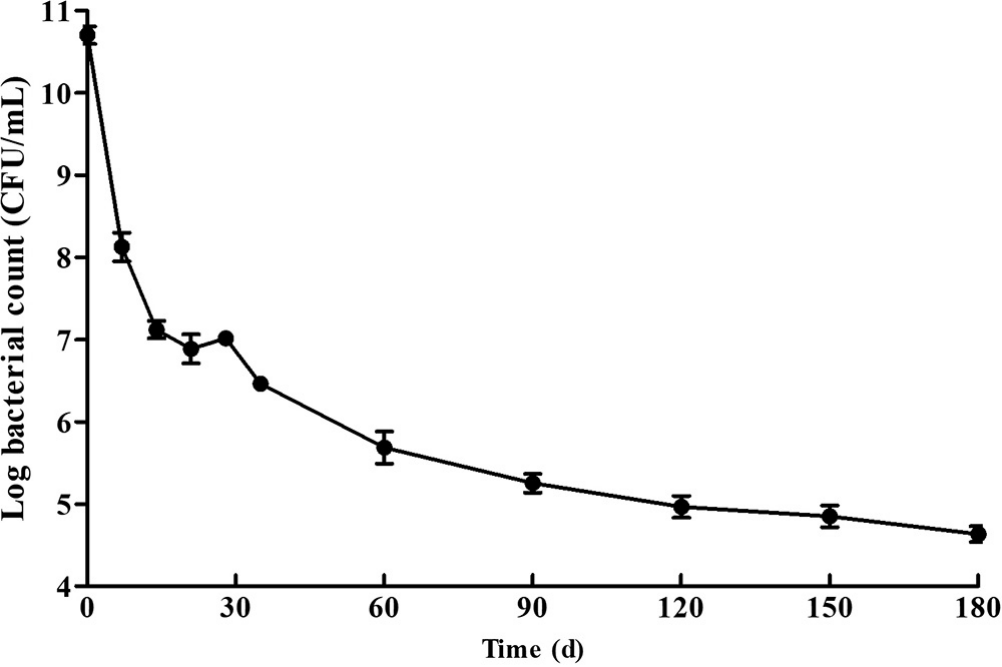
Survival curve of non-O1/O139 V. cholerae GXFL1-4 cells under starvation stress.

The survival of non-O1/O139 V. cholerae strain GXFL1-4 under starvation is shown in Fig. 1. Non-O1/O139 V. cholerae GXFL1-4 was still culturable after 6 months of starvation. The cell counts continued to decline until day 21, with the CFU/mL converted to base 10 logarithm changing from 10.71 ± 0.03 (mean ± standard deviation) to 6.92 ± 0.14, followed by a rebound at day 21. Then, the cell counts declined without interruption to 4.63 ± 0.10 by the end of storage at day 180.

### Phenotype changes of non-O1/O139 V. cholerae after starvation stress.

**FIG 2:**
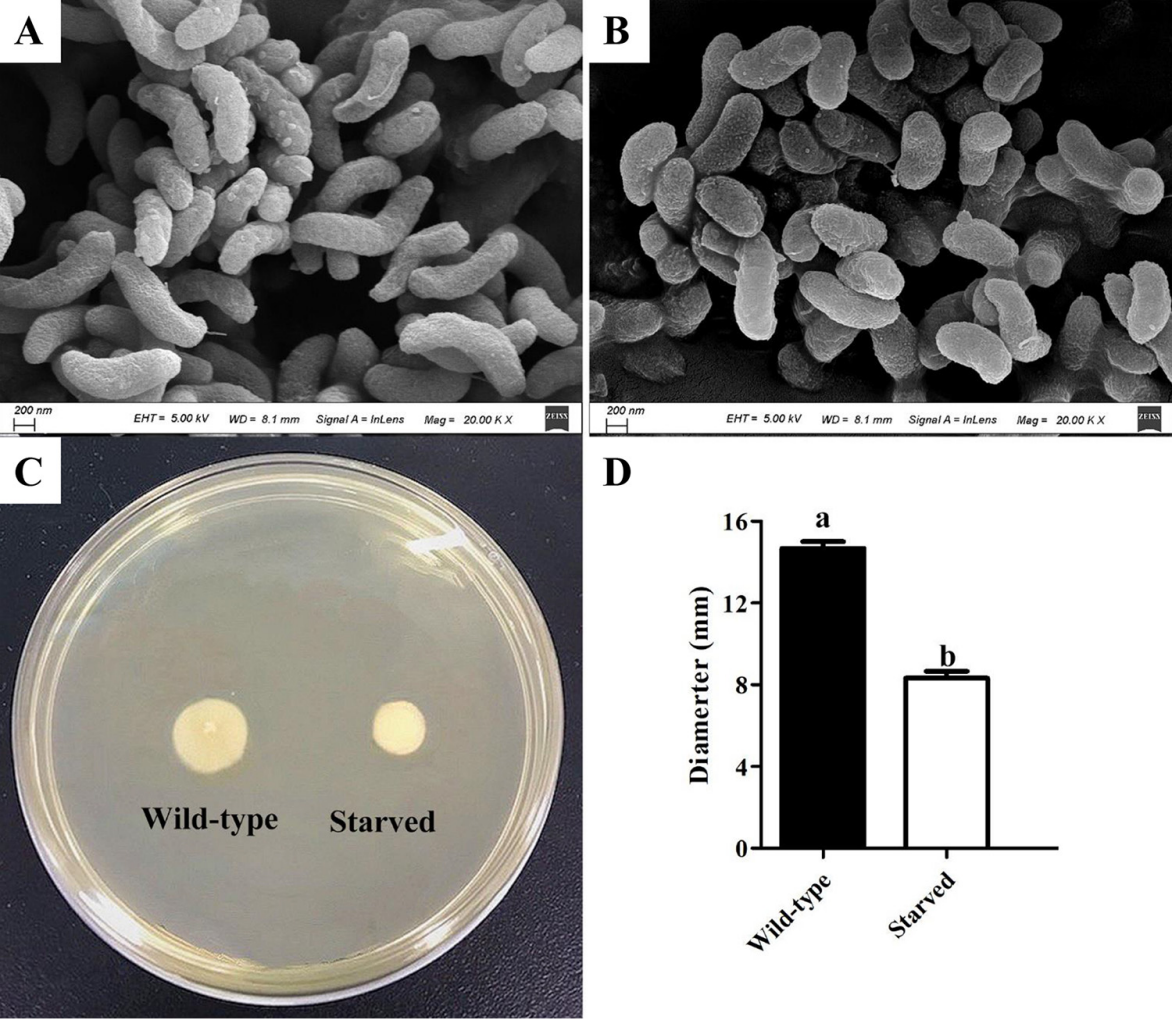
Phenotype changes of non-O1/O139 V. cholerae cells after starvation. (A) Morphology of wild-type cells revealed by scanning electron microscopy. (B) Morphology of starved cells by scanning electron microscopy. (C) Motility of starved cells after starvation. (D) Migration diameters of starved and wild-type cells. Values marked with different letters (a, b) are significantly different (*P < *0.05). Error bars show standard deviations.

**FIG 3:**
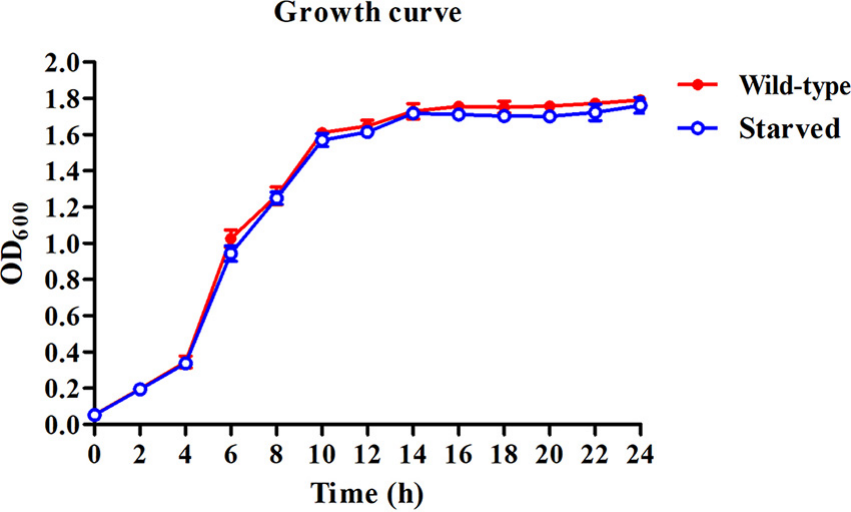
Growth curves of starved and wild-type non-O1/O139 V. cholerae. Data are presented as mean values ± SD from three independent biological replicates.

The morphologies of 6-months-starved and wild-type cells were examined using scanning electron microscopy (SEM). The starved cells had decreased significantly in size (from 2.2 ± 0.08 μm to 1.4 ± 0.16 μm in length) and exhibited shortened rod shapes, shrinking, and a rougher surface (Fig. 2A and B). Furthermore, the motility of non-O1/O139 V. cholerae cells decreased after starvation (Fig. 2C and D), while the growth curves showed that the 6-months-starved strain displayed almost the same growth rate as the wild type, indicating that the reduced motility was not due to a slower growth phenotype of the starved cells (Fig. 3). In addition, the cells showed no changes in extracellular enzymes and hemolysin activities after starvation for 6 months, and both 6-months-starved and wild-type cells could produce caseinase, lipase, amylase, lecithinase, and hemolysin activities (Table 1).

**TABLE 1:** Extracellular enzyme and hemolysin activities of non-O1 V. cholerae GXFL1-4 after starvation

Extracellular product	Mean diam (mm) ± SD (*n* = 6) of lytic zones of:	
	Wild-type cells	Starved cells
Caseinase	18.8 ± 1.8	16.0 ± 1.5
Lipase	21.8 ± 1.7	21.3 ± 1.9
Amylase	8.8 ± 1.5	6.5 ± 1.0
Lecithinase	17.8 ± 2.1	16.2 ± 0.8
Hemolysin	21.3 ± 1.6	19.0 ± 1.4

### Detection of virulence genes in starved and wild-type cells.

The results of PCR amplification of virulence-related genes in starved and wild-type non-O1/O139 V. cholerae showed that both 6-months-starved and wild-type cells were positive for *vasA*, *vasK*, *vasH*, *ompU*, *hlyA*, *rtxA*, *stn*, *mp*, and *rtxC* and negative for *zoe* and *tcpA* (Fig. S1 in the supplemental material).

### Pathogenicity of starved cells.

Comparison of pathogenicity between 6-months-starved and wild-type non-O1/O139 V. cholerae showed that starved cells presented a degree of virulence similar to that of wild-type cells (Table 2). The 50% lethal doses (LD_50_s) of starved and wild-type cells in M. rosenbergii shrimps were 7.589 × 10^6^ CFU/mL and 1.881 × 10^7^ CFU/mL, respectively.

**TABLE 2:** Pathogenicity of starved and wild non-O1 V. cholerae GXFL1-4 to M. rosenbergii shrimps

Bacterial cells	No. of shrimps	Bacterial concn (CFU/mL)	No. of dead shrimps during infection on day:							Total no. of dead shrimps	% mortality
			1	2	3	4	5	6	7		
Wild type	20	2.4 × 10^8^	14	6	0	0	0	0	0	20	100
	20	2.4 × 10^7^	4	4	3	1	0	0	0	12	60
	20	2.4 × 10^6^	3	2	1	0	0	0	0	6	30
	20	2.4 × 10^5^	2	1	0	0	0	0	0	3	15
	20	2.4 × 10^4^	0	0	0	0	0	0	0	0	0

Starved	20	2.4 × 10^8^	13	7	0	0	0	0	0	20	100
	20	2.4 × 10^7^	4	3	3	1	0	0	0	11	55
	20	2.4 × 10^6^	3	2	1	0	0	0	0	6	30
	20	2.4 × 10^5^	2	1	0	0	0	0	0	3	15
	20	2.4 × 10^4^	0	0	0	0	0	0	0	0	0

Control	20	0	0	0	0	0	0	0	0	0	0

### Transcriptome revealed gene expression related to starvation stress.

**FIG 4:**
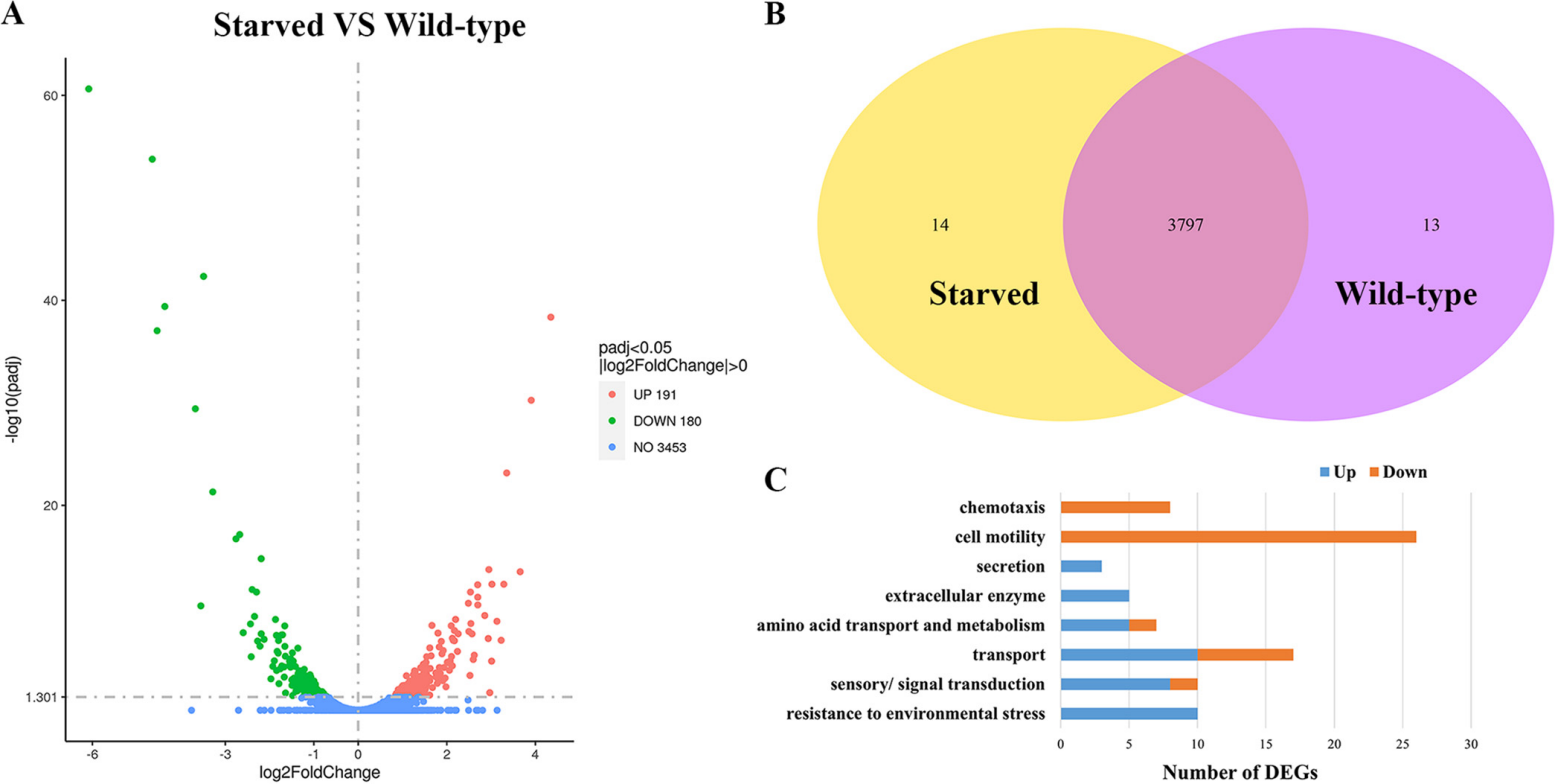
Differentially expressed genes (DEGs) of non-O1/O139 V. cholerae in starved and wild-type cells. (A) Volcano plot of DEGs. Red circles represent upregulated genes, green circles represent downregulated genes, and blue circles indicate no DEGs. (B) Venn diagram showing the overlap of gene expression levels in starved and wild-type cells. (C) DEGs involved in response to starvation based on the transcriptome analyses.

To investigate the molecular mechanisms involved in the adaptive response to starvation of non-O1/O139 V. cholerae, transcriptome profiles of the starved and wild-type cells were analyzed using RNA sequencing. After filtering through the raw reads, totals of 7,544,546, 7,753,118, and 6,675,902 clean reads from starved cells and 7,598,080, 7,307,834 and 8,211,412 clean reads from wild-type cells were obtained (Table S1). A total of 371 differentially expressed genes (DEGs) in the starved cells in comparison with the wild-type cells were identified, including 191 upregulated genes and 180 downregulated genes (Fig. 4A); the expression levels of 14 genes changed only in the starved cells and those of 13 genes changed only in the wild type (Fig. 4B). In addition, the results from gene expression difference analysis showed that large-scale DEGs were related to resistance to environmental stress, cell motility, chemotaxis, sensory/signal transduction, amino acid transport and metabolism, secretion, and transport, except for groups with unknown function and general function prediction (Fig. 4C, Table S2). Among DEGs, genes responsible for resistance to environmental stress, such as those encoding sigma factors (*rpoS*, *rpoD*, *rpoN*, and *rpoE*), universal stress proteins (*uspA* and *uspC*), cold shock proteins (*cspD*), and heat shock proteins (*hslJ*), were upregulated significantly in the starved cells, which may be involved in the response to starvation. In addition, genes responsible for flagellar assembly, such as *flgB*, *flgC*, and *flgD*, were downregulated significantly in the starved cells, which may improve the survival of non-O1/O139 V. cholerae by reducing energy requirements.

### GO and KEGG analysis of DEGs involved in response to starvation.

**FIG 5:**
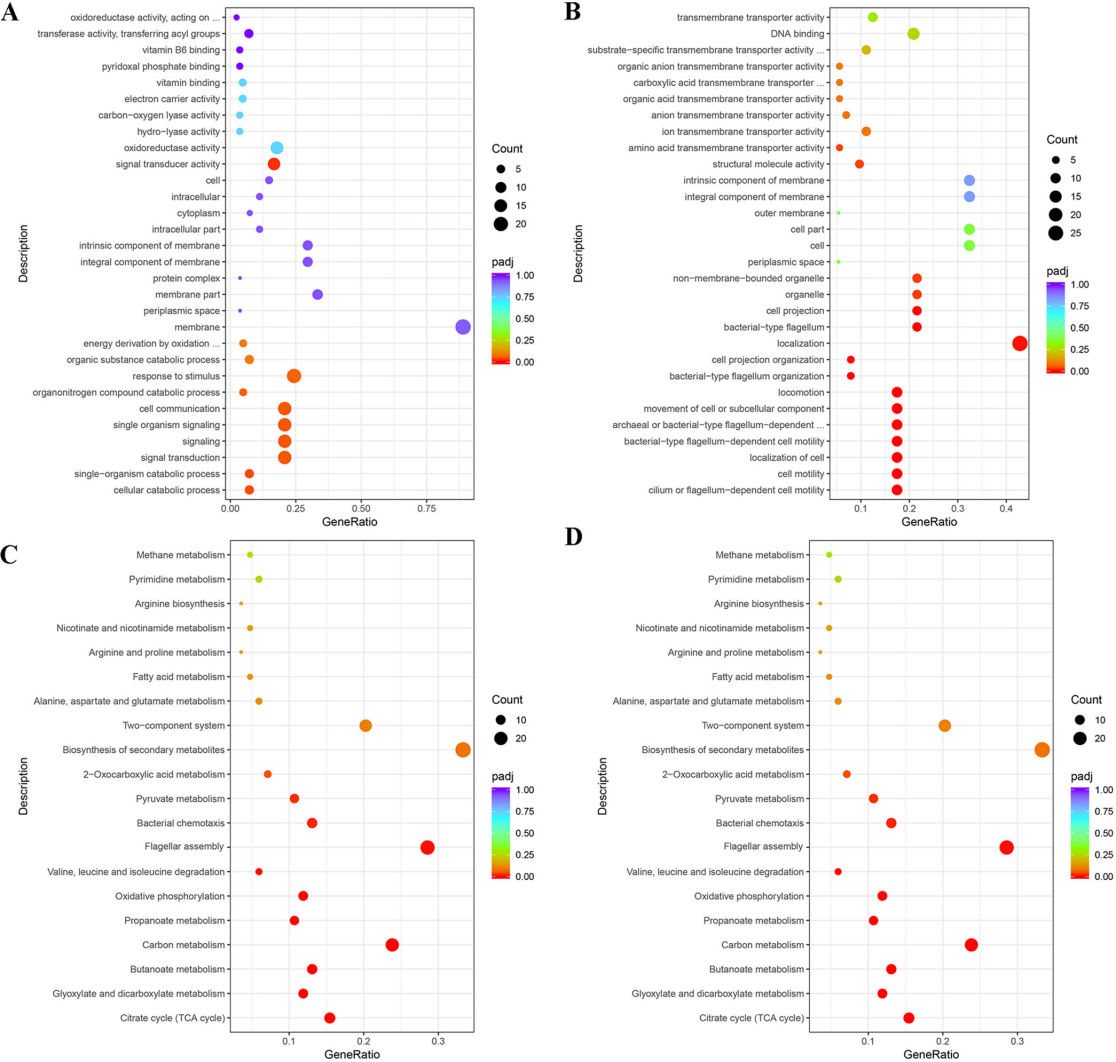
GO and KEGG analyses of genes differentially expressed between the 6-months-starved and wild-type non-O1/O139 V. cholerae strains. (A) Upregulated GO terms. (B) Downregulated GO terms. (C) Upregulated KEGG pathways. (D) Downregulated KEGG pathways.

Genes that were differentially expressed between 6-months-starved and wild-type cells were classified by using Gene Ontology (GO) software tools, and they were classified into three main categories (cellular component, molecular function, and biological process). The top three categories in the biological process category were signal transduction, signaling, and single organism signaling. The two most enriched terms in the cellular component category were cell and cell part. The most abundant subcategories in the molecular function category were ion transmembrane transporter activity, substrate-specific transmembrane transporter activity, and signal transporter activity. Among these classifications, the environmental adaptation-related subcategories, such as response to stimulus, single organism signaling, signal transduction, and signal transducer activity, were upregulated significantly in the starved cells (Fig. 5A), while bacterial-type flagellum-dependent cell motility and cell motility were downregulated (Fig. 5B).

**FIG 6:**
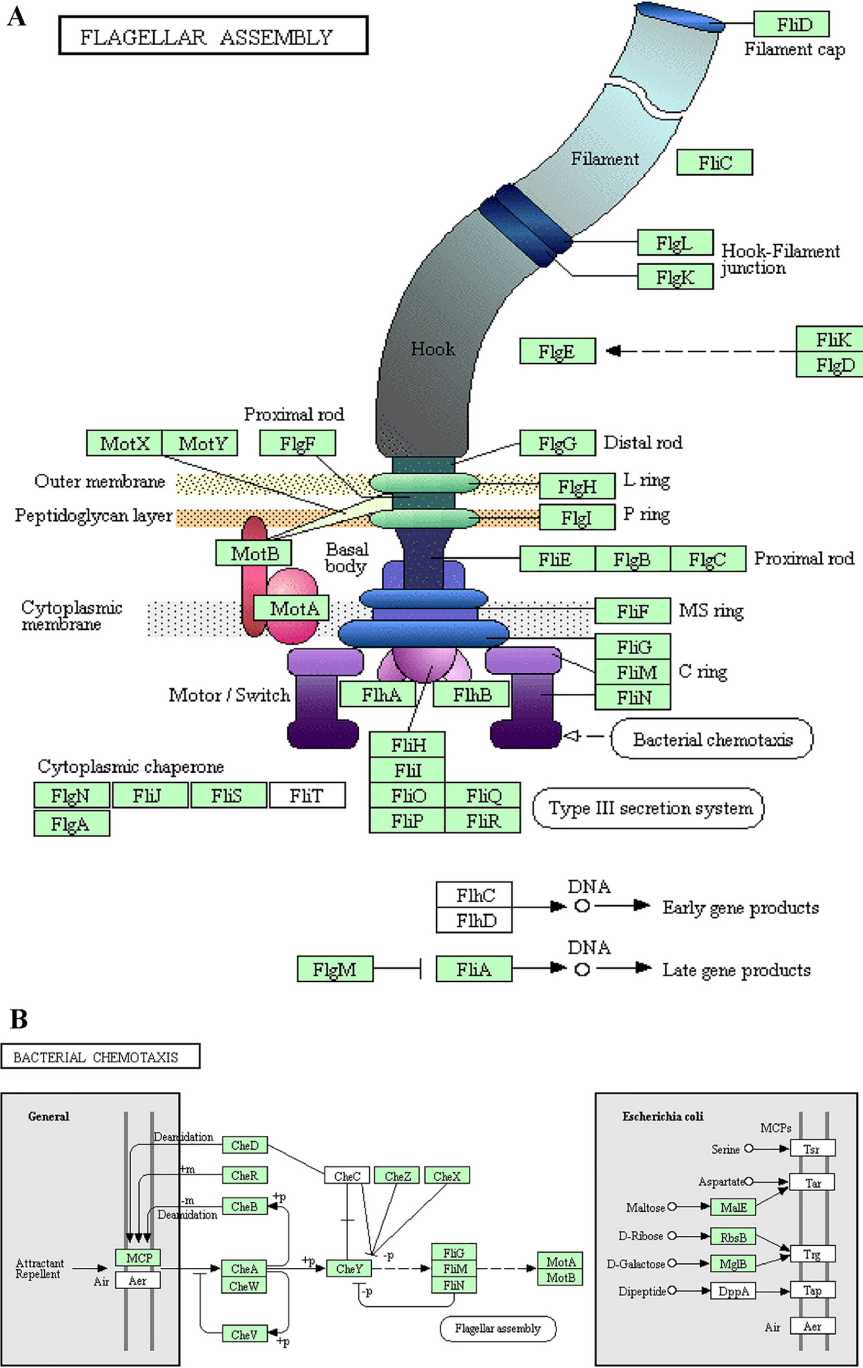
Pathway maps of flagellar assembly (A) and bacterial chemotaxis (B) pathways in KEGG. Green boxes represent downregulated genes.

A total of 54 pathways were significantly enriched in KEGG (Kyoto Encyclopedia of Genes and Genomes) analysis. The upregulated genes in starved cells were mainly clustered into microbial metabolism in diverse environments, biofilm formation, two-component system, and quorum sensing stress resistance related pathways (Fig. 5C), while the genes related to environmental adaptation (*rpoS*, *rpoD*, *rpoN*, *rpoE*, *uspA*, *uspC*, *cspD*, *hslJ*, *luxO*, etc.) were upregulated significantly in non-O1/O139 V. cholerae after starvation. The downregulated genes in starved cells were mainly clustered into flagellar assembly (*flgB*, *flgC*, *flgD*, etc.) and bacterial chemotaxis (*cheA*, *cheB*, *cheD*, *cheW*, etc.) (Fig. 5D and 6).

### Verification of the DEGs by qRT-PCR.

Eight DEGs were selected for validation of the transcriptome results. The quantitative real-time PCR (qRT-PCR) results exhibited similar expression tendencies as in transcriptome analysis, which confirmed the reliability of the transcriptome sequencing results (Fig. S2).

## DISCUSSION

Although it is widely accepted that the adaptive capacity in response to environmental stress is one of the most essential reasons for pathogenic bacteria to cause diseases of aquatic animals, the survival strategies of non-O1/O139 V. cholerae in the aquatic environment are not well understood. Previous studies showed that Vibrio spp. had a long-term-survival ability in water with low nutrient conditions (8, 9, 13). To study the physiological response and the long-term survival mechanism upon starvation stress is urgent and important. To address this issue, we have studied the survival characteristics and molecular-level responses of non-O1/O139 V. cholerae under starvation stress.

In natural environments, bacteria are expected to experience repeated starvation, which induces a strong response in bacterial cells and markedly influences their physiology and morphology (14). In this study, the survival and morphology of non-O1/O139 V. cholerae cells were investigated under starvation conditions, and the results showed that non-O1/O139 V. cholerae could survive a long time of 6 months with reduction in the size of cells, turning them from rods to approximately spherical, which was consistent with the long-term survival and morphological changes of other Vibrio spp. (15, 16). It is clear that bacteria have evolved mechanisms to survive under starvation stress by reducing cell size and turning into a round cell shape (17). Furthermore, some bacteria adopt a survival strategy under starvation stress of entering into a viable-but-nonculturable (VBNC) state (18). It has been suggested that these responses reflect the existence of various kinds of strategies to enhance survival under conditions of exogenous nutrient deprivation.

Bacteria are not only able to counteract the starvation stress by adopting survival strategies, but also retain their virulence to the host (19). Stable virulence of bacteria under starvation stress was reported previously. Ben Kahla-Nakbi et al. (20) reported that long-starved cells of Vibrio alginolyticus maintained their infectivity for gilthead sea bream and sea bass. Similarly, Sundberg et al. (21) reported that starved Flavobacterium columnare had significantly higher virulence to fish than the wild type. In our study, the 6-months-starved cells maintained their infectivity to M. rosenbergii shrimps, suggesting that non-O1/O139 V. cholerae can undergo a rapid adaptation to nutrient deficiency and express virulence to aquatic animals. Moreover, the detection of virulence factors showed that the starved non-O1/O139 V. cholerae cells still produced caseinase, lipase, amylase, lecithinase, and hemolysin activities, supporting the virulence of the starved non-O1/O139 V. cholerae cells to aquatic animals. Vibrio species are known to produce various extracellular products, including caseinase, lipase, amylase, and lecithinase, which are mainly involved in providing peptide nutrients for the microorganism (22). In addition, the detection of virulence-related genes showed that the starved cells still possessed *vasA*, *vasK*, *vasH*, *ompU*, *rtxA*, *hlyA*, *stn*, *mp*, and *rtxC*. Previous studies showed that the mRNA quantities of many virulence-related genes in starved Vibrio cells remained stable (22). Therefore, knowing the expression of genes in bacteria responding to environmental stress is important for understanding the molecular adaptations for survival and pathogenesis.

To further identify the survival mechanisms of non-O1/O139 V. cholerae under starvation stress, transcriptome analyses of starved and wild-type non-O1/O139 V. cholerae cells were conducted. In the present study, changes of gene expression in non-O1/139 V. cholerae cells in response to starvation were investigated by transcriptome sequencing (RNA-Seq), and sigma factors (*rpoS*, *rpoD*, *rpoN*, and *rpoE*) were found to be upregulated significantly in the starved cells. Previous studies showed that bacteria possessed many sigma factors that would be helpful in combating environmental stress (23), and RpoS is one of the most important transcriptional regulators for stress, controlling the expression of different stress response pathways (24). In addition, GO and KEGG enrichment analyses were used to analyze the differential expression of genes in response to starvation. In GO analysis, transcripts were clustered into the environmental adaptation related subcategories of response to stimulus, single organism signaling, signal transduction, and signal transducer activity, which may be involved in the defense and resistance of non-O1/139 V. cholerae toward nutrient deficiency. In KEGG pathways, there were many significantly enriched pathways, such as metabolism in diverse environments, biofilm formation, two-component system, quorum sensing, flagellar assembly, bacterial chemotaxis stress resistance related pathways, etc., which were also closely related to starvation stress (25, 26). Previous studies showed that two-component systems were systems consisting of two conserved proteins and were crucial in maintaining the bacterial response to environmental stresses (27). Similarly, Alcántara et al. (28) reported that two-component systems played a major role in the physiology of Lactobacillus casei and its adaptation under changing environmental conditions. Quorum sensing not only responded to bacterial density changes but also could react to environmental stress; e.g., Joelsson et al. (29) reported that the quorum-sensing signaling system acting through HapR enhanced the expression of *rpoS* and resistance to various stresses, consistent with upregulated expression of *rpoS* in starved non-O1/O139 V. cholerae cells. In this study, we also found that flagellar assembly and bacterial chemotaxis KEGG pathways were downregulated significantly in starved cells. Previous studies showed that flagellar assembly needs a large amount of matter and energy (26), and this pathway was also sensitive to environmental stresses (30), which explains why the flagellar assembly pathway was downregulated significantly in our study. Therefore, starved bacteria will adjust their gene expression to adapt to environmental stress.

In conclusion, long-term-starved cells of non-O1/139 V. cholerae, which exhibited reduction in size, were still viable and maintained virulence to aquatic animals. The transcriptome sequence analysis proposed that non-O1/139 V. cholerae cells could survive under starvation stress by upregulating or downregulating a series of stress resistance-related genes. Overall, this work provides a broader understanding of the long-term survival of non-O1/139 V. cholerae under the starvation stresses of the aquatic environment, which eventually may allow improved measures to prevent the dissemination of this devastating pathogen in aquaculture.

## MATERIALS AND METHODS

### Bacterial strain and starvation stress.

The non-O1/O139 V. cholerae strain GXFL1-4 used in this study was isolated from diseased M. rosenbergii shrimps (3). Strain GXFL1-4 was inoculated into 5 mL of LB liquid medium and incubated at 28°C for 18 h with shaking. Five milliliters of the culture was inoculated into 100 mL of LB liquid medium and incubated at 28°C for 18 h with shaking. For starvation experiments, the culture was centrifuged at 8,000 × *g* for 10 min at 4°C, and the pellet was washed three times with sterile saline solution. Then, the cells were resuspended into Erlenmeyer flasks containing 100 mL of sterile seawater, stored at room temperature, and monitored for a period of 6 months. Three independent replicates were conducted for statistical analysis.

### Cell viability and enumeration.

The cells were sampled at day 1, day 7, day 14, day 21, day 28, day 35, day 60, day 90, day 120, day 150, and day 180 for CFU counts. Plate counts of cultivable cells were determined by the plate count method (10), using LB agar medium. The plates were incubated at 28°C for 24 h.

### Electron microscopy.

Changes in morphology between 6-months-starved and wild-type cells were monitored using scanning electron microscopy (SEM). Briefly, the cells were fixed in 2.5% glutaraldehyde solution for 24 h and dehydrated in a graded series of ethanol solutions for critical-point drying in carbon dioxide. The specimens were coated with gold for observation under a Zeiss EVO 50 electron microscope (Zeiss, Germany).

### Growth curves.

Overnight cultures of starved and wild non-O1/O139 V. cholerae were adjusted to an optical density at 600 nm (OD_600_) of 0.5, diluted 1:100 into LB medium, and cultivated at 28°C. The OD_600_ values were recorded every 2 h for 24 h, and then the growth curves were plotted, comparing the starved and wild-type non-O1/O139 V. cholerae.

### Motility assay.

The concentration of overnight-cultured 6-months-starved and wild-type cells was adjusted to an OD_600_ of 0.3 with sterile saline solution. Amounts of 1 μL of the bacterial suspensions were spotted into the center of LB plates with 0.4% agar, and the plates were incubated at 28°C for 18 h, after which the diameters of the colonies were measured and recorded.

### Determination of extracellular enzymes and hemolysin.

The concentration of overnight-cultured 6-months-starved and wild-type cells was adjusted to an OD_600_ of 0.5 with sterile saline solution, and amounts of 5 μL of the bacterial suspensions were spot inoculated onto LB nutrient agar that contained 1% gelatin (gelatinase test), 1% Tween 80 (lipase test), 2.5% skim milk (caseinase test), 2% starch (amylase test), 10% egg yolk (lecithinase test), or 7% rabbit erythrocytes (hemolysin test) as the substrate. These plates were incubated for 24 h to 48 h at 28°C, and the presence of the lytic halo around the colonies was observed. All tests were performed in six repetitions.

### Detection of virulence-related genes.

The 6-months-starved and wild-type non-O1/O139 V. cholerae cells were subjected to PCR assays to detect virulence-related genes, including metalloprotease (*mp*), heat-stable enterotoxin (*stn*), hemolysin (*hlyA*), toxin-coregulated pilus (*tcpA*), outer membrane protein (*ompU)*, repeat in toxin (*rtxA* and *rtxC*), and type VI secretion system (*vasA*, *vasK*, and *vasH*) genes. The specific primers used are shown in Table S3.

### Virulence assays.

The pathogenicity of 6-months-starved and wild-type cells was tested in healthy M. rosenbergii shrimps reared in aquariums containing 80 L of freshwater supplemented with oxygen. The starved and wild-type cells were incubated in LB liquid medium at 28°C for 24 h, respectively. The test group shrimps were infected by exposure to 1.8 × 10^4^, 1.8 × 10^5^, 1.8 × 10^6^, or 1.8 × 10^7^ CFU/mL of starved or wild-type cells, respectively, while the control group shrimps were cultured in fresh water without any bacterial inoculation. The mortality rates of infected shrimps were monitored for 7 days, and the LD_50_s were calculated using the method described by Zhang et al. (31).

### Transcriptome sequencing and data analysis.

Samples of 6-months-starved and wild-type cells (*n* = 3) were sent to Novogene Co., Ltd., Beijing, and subjected to total-RNA isolation and cDNA preparation, where the HiSeq 2500 platform was used for transcriptome sequencing. To ensure the accuracy of the subsequent biological information analysis, the clean reads were acquired from the raw data by removing the adaptors, poly-N, and low-quality reads. Then, the high-quality clean reads were compared with the specified reference genome by using Bowtie software. Gene expression profiling was based on the fragments per kb per million reads (FPKM) method. The differentially expressed genes (DEGs) between starved and wild-type cells were determined by using the DESeq R package, with |log_2_(FoldChange)|> 0 and padj < 0.05 as selecting conditions. The Gene Ontology (GO) database was used to classify the functions of DEGs, and GO software tools were used for functional enrichment analysis (32). Kyoto Encyclopedia of Genes and Genomes (KEGG) pathway enrichment analysis was used to identify significantly enriched metabolic pathways or signal transduction pathways in DEGs compared with the whole-genome background (33).

### qRT-PCR analysis.

To validate the reliability of our transcriptome sequencing data, a total of 8 differentially expressed stress resistance-related genes were selected randomly for qRT-PCR analysis, using the same RNA samples as for the RNA-Seq profiling; the primers are listed in Table S3. The 16S rRNA gene was used as the reference gene, and the relative levels of gene expression were determined using the cycle threshold (2^−ΔΔ^*^CT^*) method.

### Ethics statement.

All treatments of shrimps in this study were strictly in accordance with the guidelines of the Animal Experiment Ethics Committee of Yangzhou University. The protocol was approved by the Animal Experiment Ethics Committee of Yangzhou University.

### Data availability.

The RNA-sequencing data were deposited in the NCBI with BioProject accession number PRJNA773998. Raw sequencing data were deposited in the Short Read Archive (SRA) of the NCBI with accession numbers SRR16553061, SRR16553060, SRR16553059, SRR16553058, SRR16553057, and SRR16553056.
