# Supplementary material for: Survival Characteristics and Transcriptomic Analyses Reveal the Adaptive Response of the Aquatic Pathogen Non-O1/O139 Vibrio cholerae to Starvation Stress
Source: Microbiol Spectr. 2022 May 9;10(3):e01939-21. doi: 10.1128/spectrum.01939-21 (PMC9241822; doi:10.1128/spectrum.01939-21)
Supplement: SUPPLEMENTAL FILE 1 — Supplemental material. Download spectrum.01939-21-s001.pdf, PDF file, 0.5 MB [file spectrum.01939-21-s001.pdf]

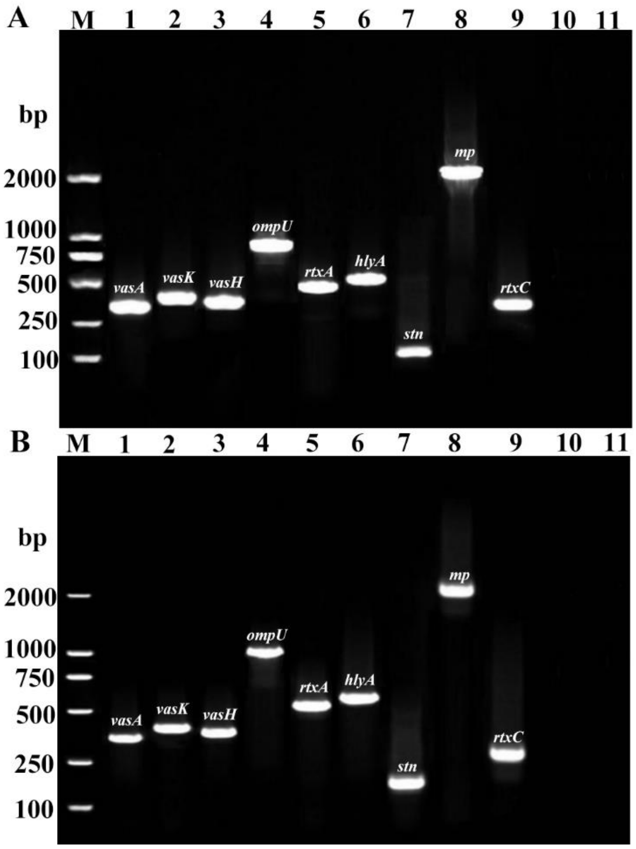

FIG S1 Detection of virulence related genes in non-O1/O139 *V. cholerae*. A: wild-type cells, B: starved cells. M, DL 2000; lane 1, *vasA*, 342 bp; lane 2, *vasK*, 399 bp; lane 3, *vasH*, 385 bp; lane 4, *ompU*, 869 bp; lane 5, *rtxA*, 417 bp; lane 6, *hlyA*, 481bp; lane 7, *stn*, 172 bp; lane 8, *mp*, 1782 bp; lane 9, *rtxC*, 265 bp. lane 10, *tcpA*, 466 bp; lane 11, *ace*, 131 bp.

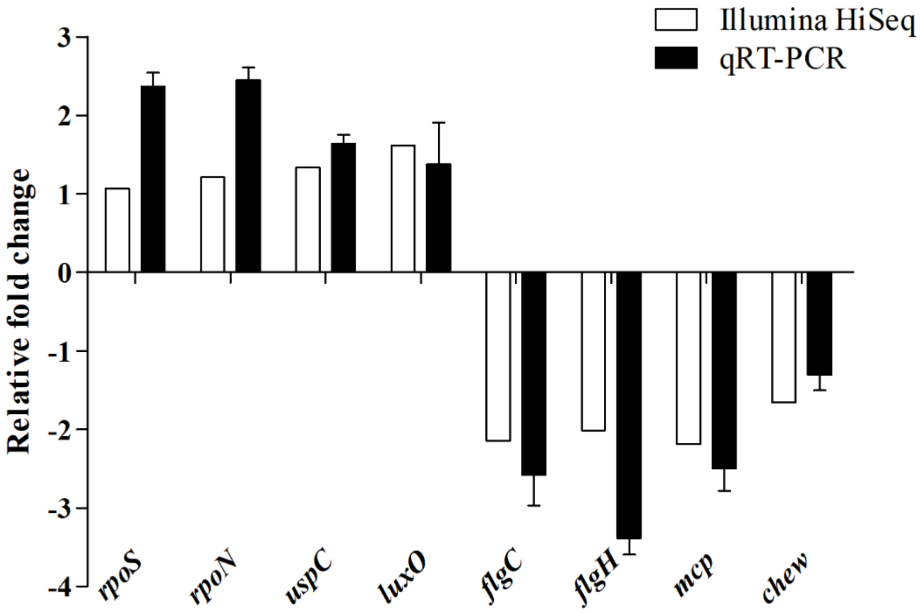

FIG S2 Comparative analysis of qRT-PCR and transcriptome sequencing result of differentially expressed genes of non-O1/O139 *V. cholerae* before and after starvation stress.

TABLE S1 Summary of transcriptome sequencing.

Sample name	Raw reads	Clean reads	clean bases	Error rate(%)	Q20(%)	Q30(%)	GC content(%)
Starved-1	7575648	7544546	1.13G	0.02	98.61	95.47	49.38
Starved-2	7813204	7753118	1.16G	0.02	98.73	95.83	49.02
Starved-3	6726900	6675902	1.0G	0.02	98.53	95.2	49.55
Wild-type-1	7639510	7598080	1.14G	0.02	98.8	96.06	49.59
Wild-type-2	7370464	7307834	1.1G	0.02	98.65	95.62	49.7
Wild-type-3	8249082	8211412	1.23G	0.02	98.74	95.88	49.72

TABLE S2 DEGs in starved non-O1 *V. cholerae* related to starvation stress.

**gene_id**	**gene_description**	**log2Fold Change**	**category**
GXFL1.4_GM003444	RNA polymerase sigma factor RpoS	1.0679	resistance to environmental stress
GXFL1.4_GM000332	RNA polymerase sigma factor RpoD	3.0191	resistance to environmental stress
GXFL1.4_GM001498	RNA polymerase sigma factor RpoN	1.2087	resistance to environmental stress
GXFL1.4_GM001555	RNA polymerase sigma factor RpoE	1.2234	resistance to environmental stress
GXFL1.4_GM002297	heat shock protein HslJ	1.2724	resistance to environmental stress
GXFL1.4_GM002297	cold shock domain-containing protein CspD	1.4590	resistance to environmental stress
GXFL1.4_GM000369	energy transducer TonB	2.5229	resistance to environmental stress
GXFL1.4_GM000349	cold-shock' DNA-binding domain	3.1313	resistance to environmental stress
GXFL1.4_GM000038	universal stress protein family UspC	1.3351	resistance to environmental stress
GXFL1.4_GM001210	universal stress global response regulator UspA	1.0222	resistance to environmental stress
GXFL1.4_GM000769	two component sensor histidine kinase	1.2449	sensory/ signal transduction
GXFL1.4_GM001343	copper-sensing two-component system response regulator CpxR	1.1188	sensory/ signal transduction
GXFL1.4_GM000325	sensor domain-containing diguanylate cyclase	1.3353	sensory/ signal transduction
GXFL1.4_GM000344	sensory box/GGDEF family protein	1.1373	sensory/ signal transduction
GXFL1.4_GM003281	sensor domain-containing diguanylate cyclase	1.0370	sensory/ signal transduction
GXFL1.4_GM003400	quorum-sensing regulator of virulence HapR	-2.39420	sensory/ signal transduction
GXFL1.4_GM002932	quorum-sensing LuxO	1.6165	sensory/ signal transduction
GXFL1.4_GM001385	quorum sensing	1.4089	sensory/ signal transduction
GXFL1.4_GM000544	molecular chaperone DnaJ	1.8728	sensory/ signal transduction
GXFL1.4_GM001122	bacterial type II and III secretion system family protein	0.8590	secretion

GXFL1.4_GM001069	leukocidin/hemolysin toxin family	1.6645	secretion
GXFL1.4_GM000420	RTX toxin-related Q Secondary metabolites biosynthesis	1.2872	secretion
GXFL1.4_GM003483	extracellular deoxyribonuclease Dns	1.5511	extracellular enzyme
GXFL1.4_GM000665	ompA family protein	1.7517	extracellular enzyme
GXFL1.4_GM002592	outer membrane protein OmpV	2.9357	extracellular enzyme
GXFL1.4_GM002525	OmpA-like transmembrane domain	1.8524	extracellular enzyme
GXFL1.4_GM003869	bacterial extracellular solute-binding proteins	1.4435	extracellular enzyme
GXFL1.4_GM001790	ABC transporter substrate-binding protein	3.9063	transport
GXFL1.4_GM003220	sodium-dependent phosphate transporter	2.5426	transport
GXFL1.4_GM000122	ABC transporters	2.0779	transport
GXFL1.4_GM003794	ABC-type antimicrobial peptide transport system ATPase component	2.0590	transport
GXFL1.4_GM000365	fecCD transport family protein	1.9625	transport
GXFL1.4_GM000661	probable anaerobic C4-dicarboxylate transporter DcuC	1.2980	transport
GXFL1.4_GM002306	divalent cation transporter	1.1626	transport
GXFL1.4_GM002392	efflux transporter, RND family, MFP subunit	1.3488	transport
GXFL1.4_GM000121	Binding-protein-dependent transport system inner membrane component	1.5068	transport
GXFL1.4_GM000793	Formate/nitrite transporter	1.1724	transport
GXFL1.4_GM001388	N-acetyl-gamma-glutamylphosphate reductase	2.5340	amino acid transport and metabolism
GXFL1.4_GM001693	acetyltransferase (GNAT) domain	2.1981	amino acid transport and metabolism
GXFL1.4_GM003233	branched-chain amino acid transport system II carrier protein	1.1426	amino acid transport and metabolism
GXFL1.4_GM000422	amino acid permease	0.9758	amino acid transport and metabolism
GXFL1.4_GM001261	threonine/homoserine/homoserine lactone efflux protein E	1.0570	amino acid transport and metabolism
GXFL1.4_GM001807	flagella basal body rod protein	-2.94924	cell motility
GXFL1.4_GM001795	flagella flgO	-2.69727	cell motility
GXFL1.4_GM001812	bacterial-type flagellum	-2.69905	cell motility
GXFL1.4_GM001810	flagellar P-ring protein FlgI	-2.48677	cell motility
GXFL1.4_GM001803	flagellar basal body rod protein FlgB	-2.85697	cell motility
GXFL1.4_GM001870	flagella fliK flagellar hook-length control protein FliK,	-2.17390	cell motility
GXFL1.4_GM002944	flagella motY sodium-type flagellar protein MotY	-2.49815	cell motility
GXFL1.4_GM001804	flagellar basal-body rod protein FlgC	-2.14628	cell motility
GXFL1.4_GM001814	flagellar motility Gene fliC	-1.87488	cell motility

GXFL1.4_GM001805	flagellar basal body rod modification protein FlgD	-1.91855	cell motility
GXFL1.4_GM001808	flagellar basal-body rod protein FlgG	-2.10701	cell motility
GXFL1.4_GM001809	flagellar basal body L-ring protein FlgH	-2.01061	cell motility
GXFL1.4_GM001813	flagellar biosynthesis protein FlgL	-1.83291	cell motility
GXFL1.4_GM001806	flagellar hook protein FlgE	-1.46135	cell motility
GXFL1.4_GM001855	flagellin D	-1.45617	cell motility
GXFL1.4_GM001794	flagella flgT flagellar protein FlgT	-1.53452	cell motility
GXFL1.4_GM001924	flagellar biosynthesis regulator FlhF	-1.47557	cell motility
GXFL1.4_GM001860	flagellar biosynthesis protein FliS	-1.36389	cell motility
GXFL1.4_GM001815	flagellin C	-1.29035	cell motility
GXFL1.4_GM001811	flagellar rod assembly protein/muramidase FlgJ	-1.41581	cell motility
GXFL1.4_GM001865	flagellar M-ring protein FliF	-1.39848	cell motility
GXFL1.4_GM001798	flagellar biosynthesis anti-sigma factor FlgM	-1.27322	cell motility
GXFL1.4_GM001854	flagellin hook IN motif family protein	-1.14299	cell motility
GXFL1.4_GM001872	flagellar motor switch protein FliM	-0.93092	cell motility
GXFL1.4_GM001864	flagellar hook-basal body complex protein FliE	-1.45760	cell motility
GXFL1.4_GM001859	flagellar rod protein FlaI	-1.33615	cell motility
GXFL1.4_GM000662	methyl-accepting chemotaxis (MCP) signaling domain protein	-1.54579	chemotaxis
GXFL1.4_GM000408	methyl-accepting chemotaxis protein McpB	-1.16444	chemotaxis
GXFL1.4_GM000154	methyl-accepting chemotaxis (MCP) signaling domain protein	-2.18983	chemotaxis
GXFL1.4_GM000200	cheW-like domain protein	-1.65842	chemotaxis
GXFL1.4_GM001028	methyl-accepting chemotaxis protein	-1.65545	chemotaxis
GXFL1.4_GM000203	methyl-accepting chemotaxis protein III	-1.48584	chemotaxis
GXFL1.4_GM002661	methyl-accepting chemotaxis (MCP) signaling domain protein	-1.29445	chemotaxis
GXFL1.4_GM000204	cheR methyltransferase	-0.97988	chemotaxis
GXFL1.4_GM002548	ABC-type polar amino acid transport system, ATPase component	-1.38375	amino acid transport and metabolism
GXFL1.4_GM000308	amino acid ABC transporter, periplasmic amino acid-binding protein	-1.01877	amino acid transport and metabolism
GXFL1.4_GM002058	TRAP-type C4-dicarboxylate transport system%2C large permease component	-1.08576	transport
GXFL1.4_GM001904	glycerol-3-phosphate ABC transporter periplasmic glycerol-3-phosphate-binding protein	-1.44752	transport
GXFL1.4_GM002128	ornithine ABC transporter ATP-binding protein HisP	-1.23533	transport

GXFL1.4_GM002585	methyl-galactoside ABC transporter substrate-binding protein	-1.21509	transport
GXFL1.4_GM002922	zinc/cadmium/mercury/lead-transporting ATPase	-1.02786	transport
GXFL1.4_GM002853	ABC transporter substrate-binding protein	-0.90262	transport
GXFL1.4_GM002911	long-chain fatty acid transport protein	-1.03700	transport
GXFL1.4_GM001954	sensor histidine kinase	-1.04128	sensory/ signal transduction

TABLE S3 PCR primers used in this study

Gene	Primer sequence (5′-3′)	Annealing (℃)
ace	TGATGGCTTTACGTGGCTTGTGATC<br>GCCTGTTGGATAAGCGGATAGATGG	58
ompU	ACGCTGACGGAATCAACCAAAG<br>GCGGAGGTTTGGCTTGAAGTAG	58
rtxA	CTGAATATGAGTGGGTGACTTACG<br>GTGTATTGTTCGATATCCGCTACG	55
hlyA	GAGCCGGCATTCATCTGAAT<br>CTCAGCGGGCTAATACGGTTTA	58
stn	TCGCATTTAGCCAAACAGTAGAAA<br>GCTGGATTGCAACATATTTCGC	58
mp	ACGTCCTCTGAATTGGTTAG<br>CTGTAACCGCGTAACATGAC	58
tcpA	CACGATAAGAAAACCGGTCAAGAG<br>GATCAGCGACAGCAGCGAAA	55
rtxC	CGACGAAGATCATTGACGAC<br>CATCGTCGTTATGTGGTTGC	55
vasA	GTACGACCGATCCTGACGTT<br>ATCTGAATGGTCGTGGCTTC	58
vasK	GCGTCAAATTCAGGAAGAGC<br>CTGTCCCAGAACCCAACTGT	58
vasH	GTGGCACGCTATTTCTGGAT<br>TTTCAGCTCACGCACATTTC	58
rpoS	CACAATGCCGATCCTGAG<br>ATAGCCAAGAAGCCCAAA	60
rpoN	ACCGATTCAATGCAAGAC<br>GAACCAATTTATCCACCAA	60
uspC	GTTCACAGCACCACATCC<br>TCATAGGTTCCGCTTCTT	60
luxO	TTTTCCGCTGTGGATGAC<br>GCTTGCGATAGATGGTTGA	60
flgC	AGTCGGTTCGTCTCAATAC<br>ATCACTTTCCACAATCCC	60
flgH	AACGGGCTCGCTGTTTAA<br>CGCTTTGGTGCTTTCATT	60
mcp	TGTCCCTGACTTATTTGACT<br>TGGCGTTATACAGCACTT	60
cheW	GTTAGAGCCTCAGACCCA<br>CACATCCATACCGACACC	60
